# Whole-Genome Characterization of Prunus necrotic ringspot virus Infecting Sweet Cherry in China

**DOI:** 10.1128/genomeA.00060-18

**Published:** 2018-03-01

**Authors:** Jiawei Wang, Ying Zhai, Dongzi Zhu, Weizhen Liu, Hanu R. Pappu, Qingzhong Liu

**Affiliations:** a Key Laboratory for Fruit Biotechnology Breeding of Shandong Province, Shandong Institute of Pomology, Taian, Shandong, China; b Scientific Observation and Experiment Station of Fruits, Huang-huai Area Ministry of Agriculture, Shandong Institute of Pomology, Taian, Shandong, China; c Department of Plant Pathology, Washington State University, Pullman, Washington, USA; d Plant Breeding and Genetics Section, School of Integrative Plant Science, Cornell University, Ithaca, New York, USA

## Abstract

Prunus necrotic ringspot virus (PNRSV) causes yield loss in most cultivated stone fruits, including sweet cherry. Using a small RNA deep-sequencing approach combined with end-genome sequence cloning, we identified the complete genomes of all three PNRSV strands from PNRSV-infected sweet cherry trees and compared them with those of two previously reported isolates.

## GENOME ANNOUNCEMENT

Prunus necrotic ringspot virus (PNRSV) is a member of the genus Ilavirus in the family Bromoviridae. As a positive-strand RNA plant virus with a tripartite genome, PNRSV causes a significant yield loss of 20 to 56% in most cultivated stone fruits, such as cherry, peach, plum, apricot, and almond (1). The PNRSV genome consists of three single-stranded positive RNAs. RNA1 and RNA2 encode the replicase proteins P1 and P2, respectively; RNA3 is bicistronic and encodes the movement protein (MP) and the coat protein (CP), the latter of which is expressed from RNA4, a subgenomic RNA (2).

Here, we report the complete genome sequence of a PNRSV China isolate, SCh-Taian, infecting sweet cherry trees. PNRSV strain SCh-Taian was detected in virus-infected sweet cherry (Prunus avium L.) trees of the cultivar ‘Red Lamp’ during a cherry-infecting virus survey (3, 4) carried out in Shandong Province, China. However, no apparent symptoms were observed in these trees infected by either PNRSV strain SCh-Taian itself or a mixture of SCh-Taian and Cherry virus A (CVA) isolate Taian (3).

The complete genome of PNRSV strain SCh-Taian was obtained using a small RNA deep-sequencing approach. The end-genome sequences of SCh-Taian were determined by overlapping reverse transcription-PCR and 5′ and 3′ rapid amplification of cDNA ends. Two samples infected with PNRSV SCh-Taian and one sample dually infected with PNRSV SCh-Taian and CVA Taian were used for total RNA extraction and small RNA library construction. Low-molecular-weight RNAs were selected via polyacrylamide gel electrophoresis, and the Illumina HiSeq 2000 platform was used for small RNA sequencing. CLC Genomics Workbench version 7.5 was used for contig assembly from the pool of clean reads. More than 15,000,000 raw small RNA sequencing reads were generated from each of the three samples. Adaptor trimming and short (<18 nucleotides [nt]) or low-quality read removal resulted in a total of 340 Mb of high-quality reads. Contigs of at least 100 bp were assembled for the PNRSV strain SCh-Taian genome after *in silico* removal of the host small RNAs. Both the PNRSV SCh-Taian and the CVA Taian genomes can be assembled using reads from the dually infected sweet cherry sample.

RNA1 of PNRSV strain SCh-Taian is 3,333 nt in length and encodes the replicase protein P1 (3,138 nt, from nt positions 31 to 3168). The 2,591-nt RNA2 encodes the replicase protein P2 (2,400 nt, from nt positions 27 to 2426). The 1,950-nt RNA3 encodes the MP (852 nt, from nt positions 176 to 1027) and the CP (681 nt, from nt positions 1102 to 1782). Interestingly, compared to two previously reported full genomes of PNRSV isolates infecting cherry trees (5, 6), strain SCh-Taian has a higher similarity with the Canadian isolate Chr3 (5) than the Chinese isolate ChrYL (6) in both RNA1 (98.92% versus 96.48%) and RNA2 (98.34% versus 93.01%) sequences, while the RNA3 sequences of SCh-Taian and ChrYL are more similar (98.10%) than those between SCh-Taian and Chr3 (95.21%). These results indicate that the evolutionary patterns of PNRSV RNA1, RNA2, and RNA3 genomes are relatively independent.

In conclusion, our results provide the genomic basis for further investigation of PNRSV interactions with sweet cherry and other viruses (e.g., CVA) during the infection process.

### Accession number(s).

The whole-genome sequences of PNRSV strain SCh-Taian have been deposited in GenBank under the accession numbers KX650617 (RNA1), KX650618 (RNA2), and KX650619 (RNA3).
